# Gluten Is Not Gluten

**DOI:** 10.3390/nu16162745

**Published:** 2024-08-17

**Authors:** Majlinda Xhaferaj, Katharina Anne Scherf

**Affiliations:** 1 Department of Bioactive and Functional Food Chemistry, Institute of Applied Biosciences, Karlsruhe Institute of Technology (KIT), 76131 Karlsruhe, Germany; 2 Leibniz Institute for Food Systems Biology at the Technical University of Munich, 85354 Freising, Germany; 3 Professorship of Food Biopolymer Systems, TUM School of Life Sciences, Technical University of Munich, 85354 Freising, Germany

**Keywords:** celiac disease, gliadin, glutenin, reference material, wheat, wheat allergy

## Abstract

Wheat gluten is responsible for the unique baking properties of wheat flour, but it also causes wheat-related disorders in predisposed individuals. Different commercially available gluten materials are commonly used for a variety of assays, but a detailed characterization of their composition is missing in many cases. This is why we aimed to provide an in-depth analysis of three commonly used gliadin and gluten materials from two different batches using gel electrophoretic and chromatographic techniques. The gliadin material did not show the typical qualitative and quantitative protein composition and does not appear to be representative of wheat gliadin. The two gluten materials had the expected protein composition, but both showed large batch-to-batch variability regarding total protein content. Since these variations result in different biochemical, immunological, and functional behaviors, it is important to analyze at least the total protein content of each material and each batch.

## 1. Introduction

Wheat gluten is responsible for the unique baking properties of wheat flour that lead to bread with high loaf volume and a porous crumb structure [1]. It is prepared by washing starch and other water-soluble components out of wheat dough. The resulting viscoelastic wet mass can be dried at temperatures below 55 °C to yield a powder with >80% of protein that regains its functional properties when water is added again [2,3,4]. On a molecular level, gluten is composed of over 100 different monomeric, oligomeric, and polymeric proteins. These are typically divided according to solubility in 60% aqueous ethanol into gliadins that are soluble and into glutenins that remain insoluble and can only be solubilized after reduction of disulfide bonds. These two fractions are subdivided based on similarities in amino acid sequences into the gluten protein types ω5-gliadins, ω1,2-gliadins, α-gliadins, and γ-gliadins as well as high- and low-molecular-weight glutenin subunits (HMW-GS and LMW-GS) [5].

Gluten can also trigger wheat-related disorders (WRD) including celiac disease and wheat allergy in predisposed individuals [6,7]. In this context, it is defined as “a protein fraction from wheat, rye, barley, oats or their crossbred varieties and derivatives thereof, to which some persons are intolerant and that is insoluble in water and 0.5 mol/L NaCl” [8]. Since gluten from rye or barley is not commercially available and wheat is consumed in much larger quantities compared to rye and barley and therefore considered to be most relevant, in vitro studies and clinical challenges to study disease mechanisms of WRD are most commonly carried out using gluten from wheat.

One frequently used material is gliadin from wheat (G3375, Sigma-Aldrich, Darmstadt, Germany), which has 67 related peer-reviewed papers listed on the website of the manufacturer, of which 51 study WRD [9]. Further, gluten from wheat (G5004, Sigma-Aldrich) is also commonly used with 59 related peer-reviewed papers, of which 36 are on WRD [10]. A search using Google Scholar with “G3375” and “gliadin” gives 125 results, and one with “G5004” and “gluten” yields 110 results (search performed on 14 July 2024), which confirms that these materials are frequently used for different purposes. The true number of papers employing these materials is likely even higher because only some authors include the respective product number. Some exemplary applications include the preparation of spiked samples to compare the results of different ELISA test kits for gluten analysis [11], coating onto ELISA plates [12], the calibration of ELISA measurements [13], the assessment of the gluten-degrading capacity of enzymes [14,15], and in vitro cell-based experiments to study WRD mechanisms [16].

Furthermore, cost-, time-, and labor-intensive clinical food challenges are often performed with different flour-based or gluten-based materials [17,18,19,20,21,22], but a detailed characterization of the flour or gluten is missing in most cases or not reported. Depending on genetic and environmental factors during wheat cultivation and subsequent processing conditions, the protein content and composition of wheat flour and gluten samples have been shown to vary substantially [23,24]. Therefore, it is essential to at least determine the crude protein content of each material to be used [22,25], but better to provide a more in-depth analysis regarding different gluten protein types and their relative molecular mass distribution. Otherwise, the results from different studies are not expected to match already because the composition of the flour or gluten is unknown and likely not comparable. One brief investigation already showed significant differences in crude protein content and composition of four commercially available gliadin and gluten materials [26], but there is no further information available, especially on batch-to-batch variability.

To fill this gap, our aim was to provide a detailed analysis using gel electrophoretic and chromatographic techniques of three different commonly used gliadin and gluten materials from two different batches. Our hypothesis is that these materials may show batch-to-batch variability regarding protein content and composition and might not be representative of wheat gluten. Based on the results, we offer guidance on whether these materials are representative in terms of composition and can be recommended for use or if caution is advisable.

## 2. Materials and Methods

### 2.1. Materials

The chemicals and reagents were of analytical grade or higher. The following wheat gliadin or gluten materials were used: gliadin from wheat (Sigma-Aldrich, Darmstadt, Germany), gluten from wheat (Sigma-Aldrich), and gluten from wheat (abcr, Karlsruhe, Germany), named gliadin_sigma_, gluten_sigma,_ and gluten_abcr_ in the following, respectively. Two different batches (batch 1 and batch 2) were available for each material, with the lot numbers SLCK9191 and SLCB1594 (gliadin_sigma_), SLCH9770 and SLCB7492 (gluten_sigma_) and 1413129 and 1466972 (gluten_abcr_). The analyses were performed shortly after sourcing to ensure that there were no effects due to prolonged storage. PWG-gliadin available from Arbeitsgemeinschaft Getreideforschung e.V. (Detmold, Germany) [27] served as a comparison.

### 2.2. Sodium Dodecyl Sulfate-Polyacrylamide Gel Electrophoresis (SDS-PAGE)

The gliadin and gluten materials (2 mg) were extracted with 1 mL of extraction buffer (293.3 mmol/L sucrose, 246.4 mmol/L Tris, 69.4 mmol/L SDS, 0.51 mmol/L EDTA, 0.22 mmol/L Brilliant Blue G-250, 0.177 mmol/L phenol red, 0.105 mmol/L HCl, pH 8.5) overnight under reducing conditions with dithiothreitol (DTT, 50 mmol/L). After shaking for 10 min at 60 °C and centrifugation (2370 rcf, 5 min, 20 °C), 10 µL of the supernatant was applied to a NuPAGE 4–12% Bis-Tris protein gradient gel (1.0 mm, 10-well, Invitrogen, Carlsbad, CA, USA) with MOPS running buffer (50 mmol/L MOPS, 50 mmol/L Tris, 3.5 mmol/L SDS, 1 mmol/L EDTA, pH 7.7) including DTT (5 mmol/L) as reducing agent [28]. The gels were run at 200 V and 115 mA according to the manufacturer’s guidelines (Thermo Scientific, Bremen, Germany) with a run time of 30 min. Staining, destaining, and fixing were conducted according to Xhaferaj et al. [29]. The gels were scanned (LAS-3000, Fujifilm, Minato, Tokyo, Japan) and the molecular masses (M_r_) of the bands were estimated based on the marker proteins by the AIDA Image Analysis software V3.27.001 (Elysia-raytest, Angleur, Belgium).

### 2.3. Modified Osborne Fractionation

The protein composition of the gliadin and gluten materials was determined according to Wieser et al. [30] and Gabler et al. [31]. The materials (20 mg) were extracted stepwise first with 2 × 1.0 mL of salt solution (400 mmol/L NaCl and 67 mmol/L Na_2_PO_4_/KH_2_PO_4_ (pH 7.6)) to obtain albumins/globulins, second with 3 × 0.5 mL of 60% aqueous ethanol (*v*/*v*) to obtain gliadins and third with 2 × 1.0 mL of glutenin extraction solution (50% (*v*/*v*) 2-propanol + 50 mmol/L Tris HCl (pH 8.0) + 2 mol/L urea + 10 mg/mL DTT) to obtain glutenins. The first two extraction steps involved vortex mixing for 2 min and magnetic stirring for 10 min followed by centrifugation (3550 rcf, 25 min, 25 °C). Glutenin extraction involved vortex mixing for 2 min and magnetic stirring for 30 min at 60 °C in a water bath followed by centrifugation as described above. The supernatants of each fraction were combined, filled up to a volume of 2 mL using the respective extraction solution, filtered (0.45 µm Whatman SPARTAN, Cytiva Europe GmbH, Freiburg im Breisgau, Germany), and used for HPLC analyses.

### 2.4. Reversed-Phase High-Performance Liquid Chromatography (RP-HPLC)

The proteins in the albumin/globulin, gliadin and glutenin fractions were separated according to hydrophobicity using a Prominence HPLC (Shimadzu, Nakagyo-ku, Kyoto, Japan) and the following parameters: stationary phase: YMC Triart Bio C_18_, 150 × 2.1 mm, 3 µm (YMC Europe GmbH, Dinslaken, Germany), mobile phase A: 0.1% trifluoroacetic acid (TFA) in bidist. water and B: 0.1% TFA in acetonitrile, flow rate: 0.5 mL/min, column temperature: 60 °C, gradient for albumins/globulins: 0–0.4 min, 0% B; 0.5 min, 20% B; 8 min, 60% B; 8.1 min, 100% B; 8.1–13 min, 100% B; 13.1–27 min 0% B and gradient for gliadins and glutenins: 0–0.4 min, 5% B; 0.5 min, 30% B; 16 min, 60% B; 16.1–22.1 min, 100% B; 22.2 min, 5% B; 22.2–35 min, 0% B, detection: UV absorbance at 210 nm. The qualitative protein profiles were compared to that of PWG-gliadin [27] and quantitation was performed using the corresponding absorbance areas with an external calibration using PWG-gliadin at a concentration of 2.5 mg/mL, as described earlier for the absolute content [31]. The albumin/globulin, gliadin, and glutenin fractions were also added up to give total extractable proteins and each fraction was expressed as a proportion relative to this sum, separately for each of the triplicates.

### 2.5. Gel Permeation High-Performance Liquid Chromatography (GP-HPLC)

The relative M_r_ distribution of proteins in the gliadin and glutenin fractions were analyzed similarly to Gabler et al. [31] using gel permeation (GP)-HPLC on a Nexera XS HPLC (Shimadzu) and the following parameters: stationary phase: BioSep-SEC-s3000 (300 × 4.6 mm, 5 µm, Phenomenex, Aschaffenburg, Germany), mobile phase A: 0.1% TFA in bidist. water and B: 0.1% TFA in acetonitrile, flow rate: 0.3 mL/min, column temperature: 22 °C, isocratic with 50% A and 50% B, detection: UV absorbance at 210 nm. The proteins cytochrome C from horse heart (12.4 kDa), carbonic anhydrase from bovine erythrocytes (29 kDa), and albumin from bovine serum (66 kDa) were used to define the integration limits. The M_r_ ranges were the following: (1) >66 kDa; (2) 66–29 kDa; (3) 29–12.4 kDa; and (4) <12.4 kDa. In each range, the area under the curve (AUC) was integrated and calculated as a percentage of the total area. The gliadin fraction was also analyzed after the addition of DTT to reduce disulfide bonds between oligomers.

### 2.6. Data Analysis

All quantitative analyses were carried out in triplicates (*n* = 3). Means and standard deviations were calculated. One-way analysis of variance (ANOVA) with Tukey’s test (*p* < 0.05) was used to determine differences between the three materials and the two batches, respectively (Origin Pro 2019b, OriginLab Corporation, Northampton, MA, USA).

## 3. Results and Discussion

To begin with, we looked for gliadin and gluten materials that are commercially available in quantities of at least 100 mg and at reasonable cost from providers of chemicals and reagents, because these are the materials that could be used for in vitro studies in the context of WRD. Further, we focused on those materials that have already been used in several studies (see Introduction). These criteria excluded some materials that were available only in quantities of 5 mg and samples from manufacturers of vital wheat gluten, that we had already investigated in-depth [24,31]. Applying these criteria narrowed the availability down to only three different gliadin and gluten materials. To assess batch-to-batch variability, we analyzed two independent batches. While this choice of materials was made on purpose, it also presents one limitation of this study. Nevertheless, we believe that the findings from this study will be transferrable to other protein reference materials that are used, e.g., in the context of food allergies [32].

### 3.1. Qualitative Profile Using SDS-PAGE

**Figure 1:**
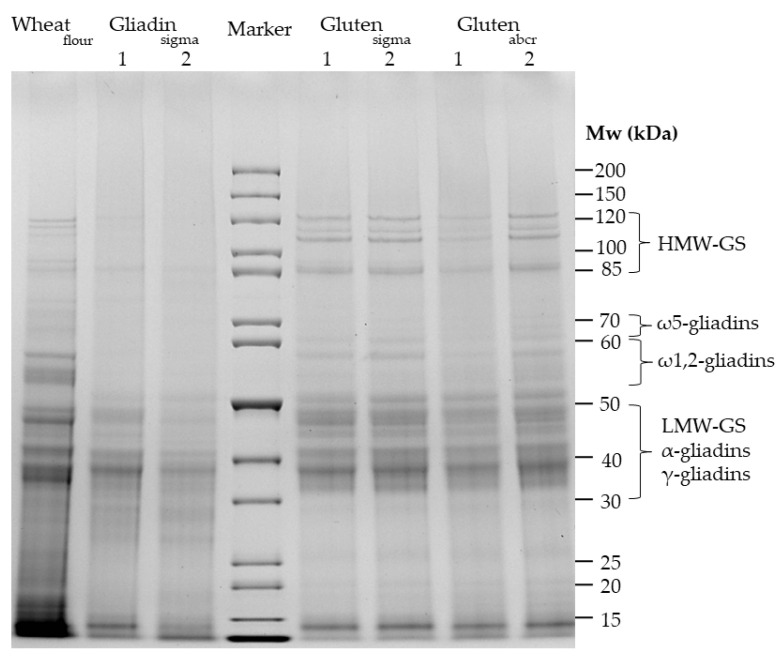
SDS-PAGE of three commercially available gliadin and gluten materials obtained from Sigma-Aldrich (sigma) and abcr (abcr). Two batches of the materials each (1 and 2) were applied to the gel and the protein extract of one representative wheat flour (cultivar Tommi).

The qualitative protein composition of the three commercially available gliadin and gluten materials was compared between both batches and to one representative wheat flour (cultivar Tommi) (Figure 1). The flour shows the typical bands with HMW-GS in the 85–120 kDa range, ω5-gliadins between 60 and 70 kDa, ω1,2-gliadins between 55 and 60 kDa and α-gliadins, γ-gliadins and LMW-GS between 30 and 50 kDa [28,33]. Gliadin_sigma_ had the smallest number of discernible bands of all materials, with only faint traces of proteins in the range above 60 kDa, indicating very low proportions of HMW-GS. According to expectations, the majority of bands were between 30 and 50 kDa, where α-gliadins and γ-gliadins, the major types within the gliadin fraction [30], are located. There were also differences between both batches of gliadin_sigma_, especially in the ranges of 40–50 kDa, 25–30 kDa, and below 15 kDa. Gluten_sigma_ and gluten_abcr_ both showed a band pattern similar to one another and to wheat flour. There were five distinct bands in the HMW-GS range in gluten_sigma_ and four in gluten_abcr_. Bands in the regions of ω5-gliadins and ω1,2-gliadins were also visible in both gluten materials, but weaker in intensity compared to the wheat flour. The majority of bands were again located in the range of α-gliadins, γ-gliadins, and LMW-GS between 30 and 50 kDa, as expected. Batches 1 and 2 were comparable in both gluten materials, respectively. All materials had bands below 30 kDa and especially below 15 kDa. These do not belong to gluten proteins, but to non-gluten proteins such as amylase/trypsin inhibitors that are part of the albumin/globulin fraction [34].

### 3.2. Qualitative Profile Using RP-HPLC

**Figure 2:**
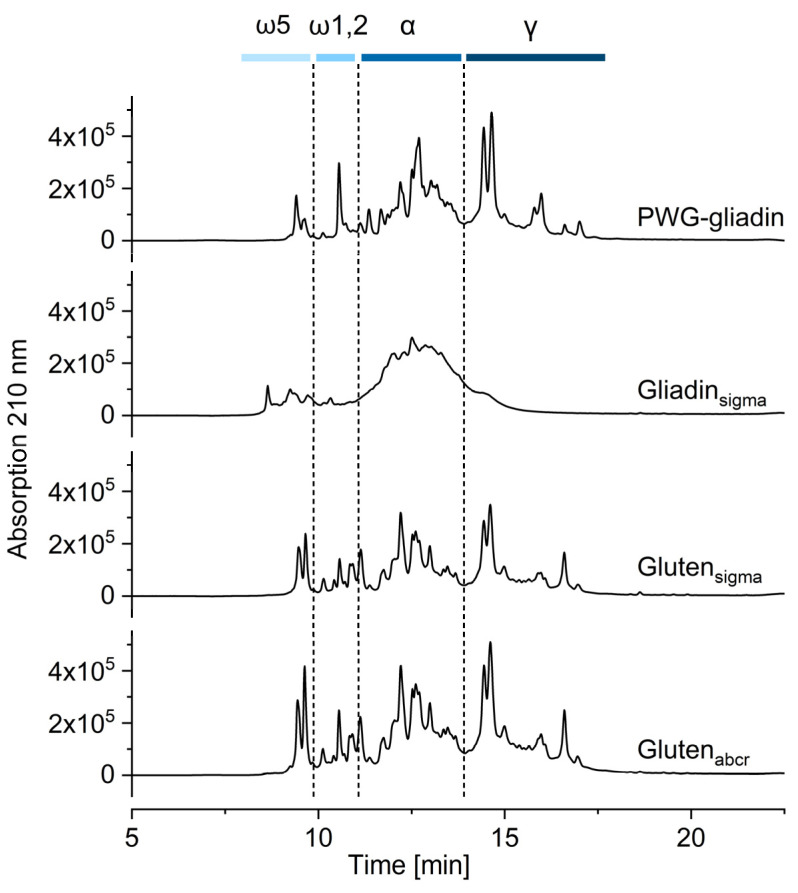
RP-HPLC chromatograms of PWG-gliadin and the gliadin fraction of three commercially available gliadin and gluten materials obtained from Sigma-Aldrich (sigma) and abcr (abcr); batch 1. The integration times are marked as dotted lines separating the wheat gluten protein types ω5-gliadins (ω5), ω1,2-gliadins (ω1,2), α-gliadins (α) and γ-gliadins (γ). For batch 2, see Figure S1.

The characteristic qualitative elution profile of PWG-gliadin was compared to the profiles of the respective gliadin fraction of gliadin_sigma_, gluten_sigma_ and gluten_abcr_. The ranges for ω5-gliadins, ω1,2-gliadins, α-gliadins, and γ-gliadins were assigned as reported before (Figure 2 and Figure S1) [30]. The gliadin fractions of both gluten materials were mostly comparable to PWG-gliadin, even if there were differences in the number of peaks, e.g., within ω1,2-gliadins. Further, there were differences in the relative height of the peaks, e.g., within ω5-gliadins, where the first peak was higher than the second one in PWG-gliadin, both were almost equal in height in gluten_sigma_ and the first peak was lower than the second one in gluten_abcr_. However, gliadin_sigma_ did not show the characteristic elution profile. Instead, there were no discernible peaks at the retention times of ω5-gliadins, ω1,2-gliadins, and γ-gliadins, and only an undefined hump where the α-gliadins are eluted. Since we could not obtain any further information on the production process of all commercially available materials, we cannot explain the exact cause of this observation. The most likely causes that are known to result in such changes in the chromatographic profile are partial chemical or enzymatic hydrolysis and/or heat treatment [31]. According to the technical support, gliadin_sigma_ is prepared following the method of Blish and Sandstedt [35]. This method involves extraction of crude gluten from flour and tap water, kneading, drying under vacuum at 60–65 °C, and grinding. The gliadin is extracted with 0.07 mol/L acetic acid, followed by filtration and treatment with LiCl for gliadin precipitation. Then, gliadin is re-dissolved in 60% ethanol and precipitated again in absolute ethanol with subsequent drying under vacuum at 50 °C. This procedure involving two drying and precipitation steps may well be the reason for the observed changes in the protein profile.

**Figure 3:**
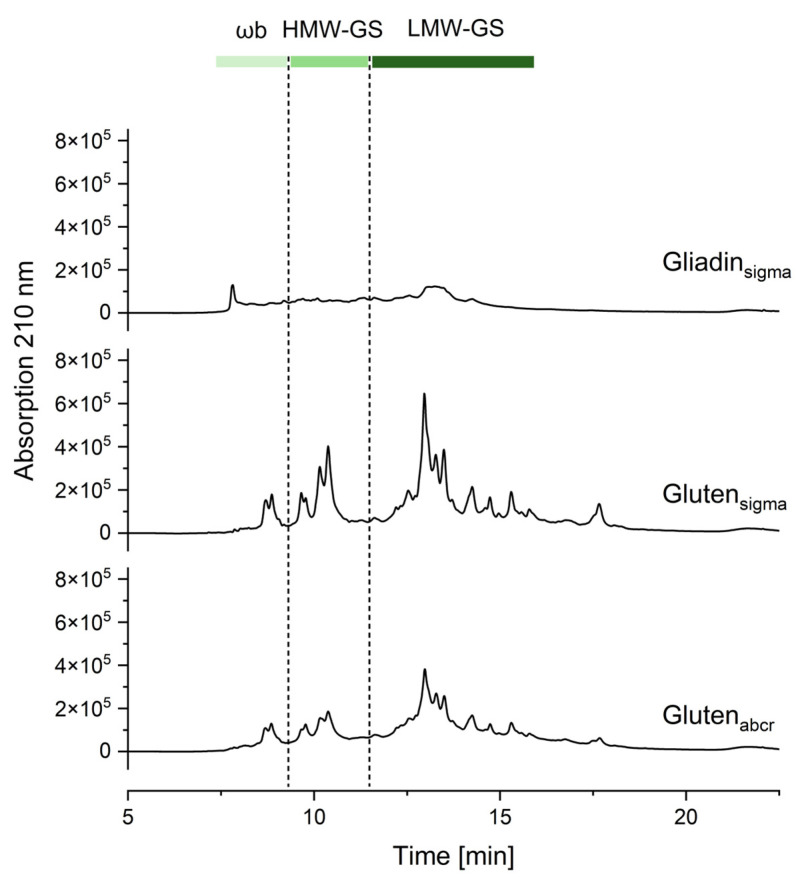
RP-HPLC chromatograms of the glutenin fraction of three commercially available gliadin and gluten materials obtained from Sigma-Aldrich (sigma) and abcr (abcr); batch 1. The integration times are marked as dotted lines separating the wheat gluten protein types ωb-gliadins (ωb), high-molecular-weight glutenin subunits (HMW-GS), and low-molecular-weight glutenin subunits (LMW-GS). For batch 2, see Figure S2.

The chromatograms of gluten_sigma_ and gluten_abcr_ showed similar elution profiles in the albumin/globulin fraction, whereas gliadin_sigma_ did not show any specific peaks. Concerning the glutenin fraction (Figure 3 and Figure S2), the chromatogram of gliadin_sigma_ showed an unspecific increase in the baseline, indicating that the material was not 100% soluble in 60% ethanol, i.e., in the gliadin fraction (see also Section 3.3). Gluten_sigma_ and gluten_abcr_ showed three major groups of peaks which are typical of ωb-gliadins, HMW-GS and LMW-GS [24,30,31]. The peaks were well separated for gluten_sigma_, whereas the peak profile of gluten_abcr_ was less clear in comparison.

### 3.3. Quantitative Protein Composition

**Figure 4:**
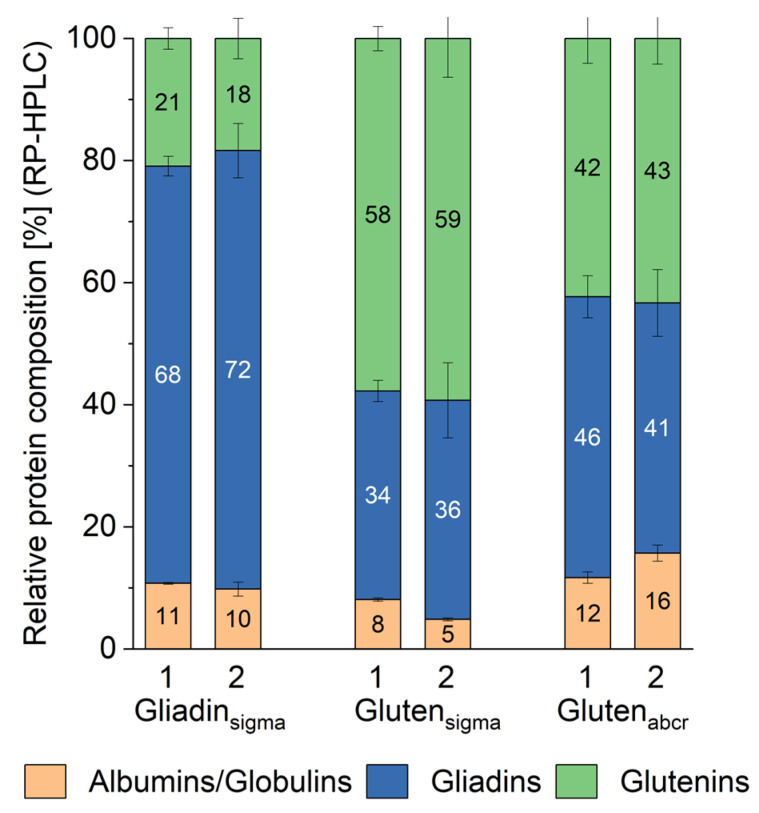
Relative protein composition of three commercially available gliadin and gluten materials obtained from Sigma-Aldrich (sigma) and abcr (abcr) shown as proportions of the three different fractions albumins/globulins, gliadins glutenins relative to total extractable proteins. Two batches each were compared. The values are given as means and the error bars indicate the standard deviation for each fraction, respectively (*n* = 3). Please see also Table S1.

The albumin/globulin, gliadin, and glutenin fractions obtained after modified Osborne fractionation were quantitated by RP-HPLC and external calibration with PWG-gliadin (Figure 4). All three materials contained albumins/globulins, gliadins, and glutenins. While we expected this for the gluten materials based on earlier findings by Gabler et al. [31], the comparatively high proportion of albumins/globulins (10–11%) and glutenins (18–21%) was remarkable for gliadin_sigma_, because the material should not contain glutenins that are insoluble in 60% ethanol. Instead, gliadin_sigma_ only contained 68–72% gliadins (Figure 4). This proportion matches the 72% of gliadins reported earlier for a different batch [26]. However, this is in contrast to PWG-gliadin, which dissolves completely in 60% ethanol [27]. There were no significant differences between the albumin/globulin, gliadin, and glutenin distribution between the two batches of gliadin_sigma_ (one-way ANOVA, *p* > 0.05, see Table S1).

Both gluten materials differed significantly in protein composition to gliadin_sigma_ and to one another (one-way ANOVA, *p* < 0.05) (Figure 4). Gluten_sigma_ was composed of 5–8% of albumins/globulins, 34–36% gliadins, and 58–59% glutenins. There were no significant differences between both batches in the proportions of gliadins and glutenins, but albumins/globulins were significantly higher in batch 1 compared to batch 2 (one-way ANOVA, *p* < 0.05). These proportions largely fit the 3% albumins/globulins, 36% gliadins, and 61% glutenins reported for a different batch of gluten_sigma_ [26]. Gluten_abcr_ was composed of 12–16% albumins/globulins, 41–46% gliadins, and 42–43% glutenins and there were no significant differences between both batches except for the albumins/globulins (one-way ANOVA, *p* > 0.05). When comparing these proportions to earlier results, we observed discrepancies, because Schwalb et al. reported 9% albumins/globulins, 56% gliadins, and 35% glutenins for a different batch [26].

The gliadin/glutenin ratios were 3.2–4.0 in gliadin_sigma_, 0.6 in gluten_sigma,_ and 0.9–1.0 in gluten_abcr_. The protein composition of both gluten materials was not comparable to the average composition of six vital wheat gluten samples G1–G6, which had 2–4% albumins/globulins, 65–69% gliadins, and 29–32% glutenins, resulting in a gliadin/glutenin ratio of 2.0–2.4 [31]. Instead, the gliadin/glutenin ratio of gluten_sigma_ (0.6) was most similar to that of G8 (0.5), which is described as a slightly textured product from wheat protein [31]. Regarding the gliadin/glutenin ratio of gluten_abcr_ (0.9–1.1), this was comparable to that of G7 (1.0), which is designated as a denatured wheat protein with high protein content [31]. Taken together, the relative protein composition of both gluten materials indicated some sort of treatment during production, most likely heating, which resulted in a decrease in gliadins and an increase in glutenins compared to typical vital wheat gluten samples that are dried using only temperatures below 55 °C [24]. These observations confirm earlier findings that recommend freeze-drying for wheat gluten preparation because vacuum drying at 60 °C for 4 h and oven drying at 80 °C for 4 h both resulted in inferior gluten properties in terms of water absorption, oil absorption, and reconstitution [36].

The gliadin and glutenin fractions were further subdivided into the gluten protein types ω5-gliadins, ω1,2-gliadins, α-gliadins, and γ-gliadins as well as ωb-gliadins, HMW-GS and LMW-GS. The values are given relative to the respective gliadin fraction (Table 1) or glutenin fraction (Table 2), with PWG-gliadin as a comparison, because its composition is representative of wheat grown in Europe [27]. All three materials differed significantly from PWG-gliadin in their composition, with more ω5-gliadins, but more or less ω1,2-gliadins, α-gliadins and γ-gliadins, depending on the material and the respective batch (Table 1). There were significant differences between batches 1 and 2 for all materials, with gluten_abcr_ showing the smallest differences between both batches. The relative composition of gliadin_sigma_ was most different from that of PWG-gliadin and also varied between both batches, especially regarding α-gliadins and γ-gliadins. While the gliadin composition of wheat flours and gluten isolates varies depending on genetics, environment, and processing [23,24,31], gliadin_sigma_ is outside typical ranges and would not be considered as representative anymore, which agrees with its atypical qualitative profile (Figure 2).

The relative glutenin composition was comparable within all three materials and the two batches (Table 2). Gliadin_sigma_ showed no discernible peaks, but integration at the same retention time intervals as for the other two materials resulted in a similar relative composition. Overall, the glutenin composition lay within typical ranges for ωb-gliadins, HMW-GS, and LMW-GS [31].

**Figure 5:**
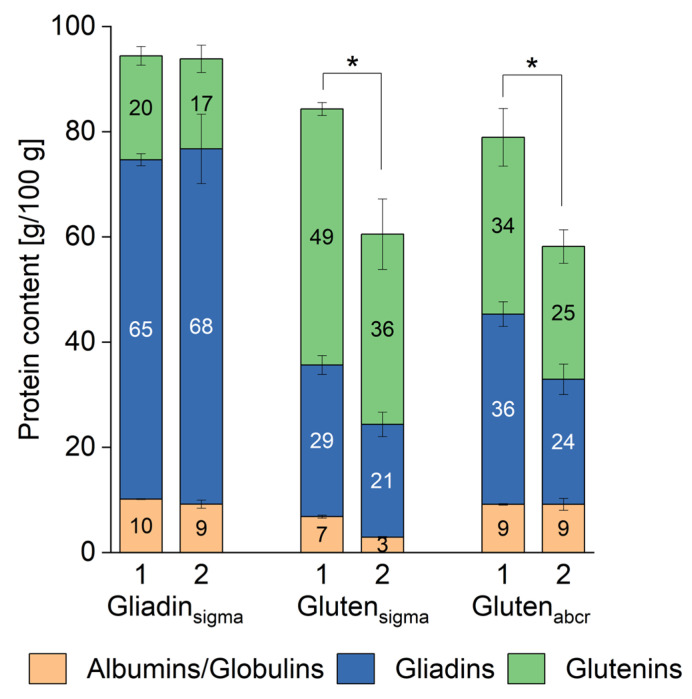
Protein composition [g/100 g] of three commercially available gliadin and gluten materials obtained from Sigma-Aldrich (Sigma) and abcr (abcr). Two batches each were compared. The values are given as means and the error bars indicate the standard deviation (*n* = 3). Significant differences between the protein content (sum of all fractions) are marked with an asterisk (one-way ANOVA, *p* < 0.05).

Considering the absolute protein content of the materials (Figure 5), gliadin_sigma_ had the highest total protein content (93.9–94.4 g/100 g) and gluten content (84.3–84.7 g/100 g) of the three materials and it was not significantly different between both batches (one-way ANOVA, *p* > 0.05). The total protein content was comparable to that of PWG-gliadin (93.1 g/100 g) [27] and to the 91.9 g/100 g reported earlier for another batch [26]. The manufacturer of gliadin_sigma_ only indicates a nitrogen content of 15 g/100 g. When using the factor of 5.7 recommended for wheat to calculate nitrogen to protein content [31], this results in 85.5 g/100 g, which is slightly lower than the content we found.

In contrast to gliadin_sigma_, there were significant differences (one-way ANOVA, *p* < 0.05) between both batches in total protein and gluten content for both gluten materials. While batch 1 of gluten_sigma_ contained 84.3 g/100 g of total protein and 77.5 g/100 g of gluten, batch 2 contained 60.5 g/100 g of total protein and 57.6 g/100 g of gluten. For gluten_abcr_, batch 1 had 79.0 g/100 g of total protein and 69.8 g/100 g of gluten, whereas batch 2 had 58.2 g/100 g of total protein and 49.0 g/100 g of gluten. The manufacturer claims a crude protein content of ≥75 g/100 g for gluten_sigma_, but we could only confirm this value for the first batch, not for the second one. There is no information on the protein content of gluten_abcr_. All values for both gluten materials are within the expected range compared to earlier studies that reported total protein content of 71.5–77.0 g/100 g [26] and 59.8–75.1 g/100 g [31], even though both batch 2 of gluten_sigma_ and batch 2 of gluten_abcr_ were at the low end or slightly below.

The most remarkable result with implications for use as a reference material was the comparatively large difference in absolute content between both batches of gluten_sigma_ and gluten_abcr_, respectively. Even though the relative composition of albumin/globulin, gliadin and glutenin fractions was mostly similar, the absolute differences are expected to have a significant impact on analytical, biochemical, immunological, and functional analyses. Therefore, it is important to analyze at least the total protein content of each material and each batch before use.

### 3.4. Relative Molecular Mass Distribution

**Figure 6:**
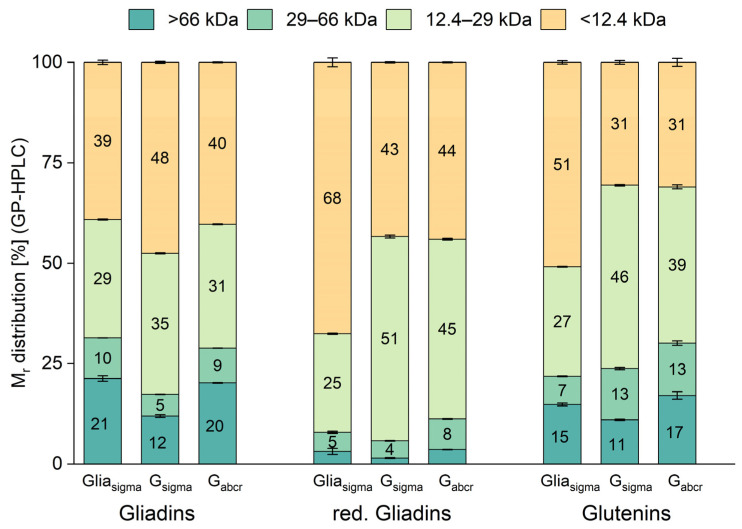
Relative molecular mass (M_r_) distribution of three commercially available gliadin and gluten materials obtained from Sigma-Aldrich (sigma) and abcr (abcr). The ranges were divided according to the following molecular masses: (1) >66 kDa, (2) 66–29 kDa, (3) 29–12.4 kDa, (4) <12.4 kDa. The values are given as means and the error bars indicate the standard deviation (*n* = 3). G: gluten, Glia: gliadin and red.: reduced. Please see also Table S2.

We also analyzed the gliadin and glutenin fractions of batch 1 of the gliadin and gluten materials by GP-HPLC to determine the M_r_ distribution in the following ranges: (1) >66 kDa; (2) 66–29 kDa; (3) 29–12.4 kDa; and (4) <12.4 kDa (Figure 6). Gliadins were additionally reduced to break disulfide bonds of oligomeric HMW-gliadins [37].

The gliadin fraction had the highest proportion in range (4), followed by (3), (1) and (2) in all three materials. The M_r_ distribution differed significantly (one-way ANOVA, *p* < 0.05) between the samples within the gliadin fraction, except for range (1) when comparing gliadin_sigma_ (21.3%) and gluten_abcr_ (20.2%). Range (1) (>66 kDa) of all three samples within the red gliadins was significantly lower compared to range (1) within the unreduced gliadin fraction. This is expected, because the reduction in the disulfide bonds leads to a disconnection of disulfide-linked gluten proteins, resulting in a decrease in HMW-gliadins [37]. For gliadin_sigma_, the reduction in disulfide bonds resulted in an increase in range (4) and a decrease in all other ranges. For gluten_sigma_ and gluten_abcr_, the proportion of range (3) increased after reduction and both gluten materials showed a largely comparable M_r_ distribution within the red. gliadins, which differed from that of gliadin_sigma_.

The M_r_ distribution of the glutenin fraction was significantly different between all samples in ranges (1) and (3). For ranges (2) and (4), both gluten materials were similar and the M_r_ distribution differed significantly from that of gliadin_sigma_ (one-way ANOVA, *p* < 0.05). The M_r_ distribution within the glutenin fraction of both gluten materials was similar to the same fraction of sample G8 [31], but the corresponding gliadin fraction of G8 did not match either of the materials studied here. Branlard et al. investigated a large wheat sample set involving 11 locations and 192 cultivars and found that the environment influenced the characteristics of glutenin polymers much more than the genetic background [38]. Therefore, the choice of the flour to be used for gluten extraction already plays an important role.

## 4. Conclusions

The detailed analysis of three different commonly used gliadin and gluten materials from two batches each using gel electrophoretic and chromatographic techniques revealed that these materials need to be characterized in-depth in terms of protein content and composition before use. Our findings thus confirmed our hypothesis concerning batch-to-batch variability regarding protein content and composition and partly poor representativeness of wheat gluten. Gliadin_sigma_ is not recommended for use, because it does not show the characteristic qualitative gliadin profile, consequently also not the typical quantitative composition of gliadins and is therefore not deemed to be representative. These changes also imply different biochemical, immunological, and functional behaviors compared to wheat flour or PWG-gliadin. Gluten_sigma_ and gluten_abcr_ showed the expected qualitative and quantitative gluten composition. However, there was a difference of up to 23 g/100 g in the absolute protein content between both batches, respectively. Therefore, at least the total protein content of each batch needs to be analyzed before use. In sum, caution is advisable when using commercially available gliadin or gluten materials, because their protein content and composition need to be analyzed before use in analytical, biochemical, functional, or immunological assays.

## Figures and Tables

**Table 1:** Relative gliadin composition of three commercially available gliadin and gluten materials obtained from Sigma-Aldrich (sigma) and abcr (abcr) shown as proportions of total gliadins. Two batches each were compared.

Sample	Batch	ω5-Gliadins	ω1,2-Gliadins	α-Gliadins	γ-Gliadins
		[%] ^1^			
Gliadin_sigma_	1	10.1 ± 0.1 ^B^	4.9 ± 0.1 ^C^	73.5 ± 0.2 ^A^	11.5 ± 0.2 ^F^
	2	11.2 ± 0.2 ^A^	5.3 ± 0.6 ^C^	54.6 ± 0.9 ^B^	28.9 ± 0.1 ^E^
Gluten_sigma_	1	8.3 ± 0.1 ^D^	5.0 ± 0.1 ^C^	46.2 ± 0.5 ^D^	40.5 ± 0.8 ^BC^
	2	9.5 ± 0.2 ^C^	13.4 ± 0.3 ^A^	41.8 ± 0.1 ^E^	35.3 ± 0.4 ^D^
Gluten_abcr_	1	8.7 ± 0.1 ^D^	5.3 ± 0.1 ^C^	46.0 ± 0.8 ^D^	40.0 ± 0.7 ^C^
	2	8.6 ± 0.1 ^D^	4.9 ± 0.3 ^C^	43.2 ± 0.2 ^E^	43.3 ± 0.5 ^A^
PWG-Gliadin		4.1 ± 0.2 ^E^	6.3 ± 0.1 ^B^	47.9 ± 0.4 ^C^	41.7 ± 0.3 ^B^

^1^ The values are given as means ± standard deviation (*n* = 3) and different capital letters indicate significant differences between the samples in each column (one-way ANOVA, Tukey’s post hoc test, *p* < 0.05).

**Table 2:** Relative glutenin composition of three commercially available gliadin and gluten materials obtained from Sigma-Aldrich (sigma) and abcr (abcr) shown as proportions of total glutenins. Two batches each were compared.

Sample	Batch	ωb-Gliadins	HMW-GS	LMW-GS
		[%] ^1^		
Gliadin_sigma_	1	13.2 ± 0.5 ^A^	30.9 ± 3.5 ^A^	55.9 ± 0.5 ^C^
	2	8.7 ± 1.0 ^B^	26.7 ± 0.5 ^A^	64.5 ± 0.3 ^B^
Gluten_sigma_	1	6.8 ± 0.2 ^CD^	23.4 ± 0.6 ^A^	69.8 ± 0.1 ^A^
	2	5.0 ± 1.1 ^D^	26.1 ± 2.1 ^A^	68.9 ± 0.9 ^A^
Gluten_abcr_	1	7.2 ± 0.4 ^BC^	23.9 ± 5.2 ^A^	68.9 ± 0.1 ^A^
	2	5.0 ± 0.9 ^D^	28.6 ± 2.4 ^A^	66.5 ± 1.4 ^B^

^1^ The values are given as means ± standard deviation (*n* = 3) and different capital letters indicate significant differences between the samples in each column (one-way ANOVA, Tukey’s post hoc test, *p* < 0.05).

## Data Availability

The original contributions presented in the study are included in the article/Supplementary Materials, further inquiries can be directed to the corresponding author.

## Supplementary Materials

The following supporting information can be downloaded at: https://www.mdpi.com/article/10.3390/nu16162745/s1, Figure S1. RP-HPLC chromatograms of the gliadin fraction of three commercially available gliadin and gluten materials obtained from Sigma-Aldrich (sigma) and abcr (abcr); batch 2. The integration times are marked as dotted lines separating the wheat gluten protein types ω5-gliadins (ω5), ω1,2-gliadins (ω1,2), α-gliadins (α) and γ-gliadins (γ). Figure S2. RP-HPLC chromatograms of the glutenin fraction of three commercially available gliadin and gluten materials obtained from Sigma-Aldrich (sigma) and abcr (abcr); batch 2. The integration times are marked as dotted lines separating the wheat gluten protein types ωb-gliadins (ωb), high-molecular-weight glutenin subunits (HMW-GS) and low-molecular-weight glutenin subunits (LMW-GS). Table S1. Relative protein composition of three commercially available gliadin and gluten materials obtained from Sigma-Aldrich (sigma) and abcr (abcr) shown as proportions of the three different fractions albumins/globulins, gliadins and glutenins relative to total extractable proteins. Two batches each were compared (see Figure 4). Table S2. Relative molecular mass (Mr) distribution of three commercially available gliadin and gluten materials obtained from Sigma-Aldrich (sigma) and abcr (abcr). The ranges were divided according to the following molecular masses: (1) >66 kDa, (2) 66–29 kDa, (3) 29–12.4 kDa, (4) <12.4 kDa (see Figure 6).
