# Supplementary material for: Gluten Is Not Gluten
Source: Nutrients. 2024 Aug 17;16(16):2745. doi: 10.3390/nu16162745 (PMC11357231; doi:10.3390/nu16162745)
Supplement: Supplementary file 1 [file nutrients-16-02745-s001.zip › nutrients-3132853-supplementary.pdf]

Supplementary Material

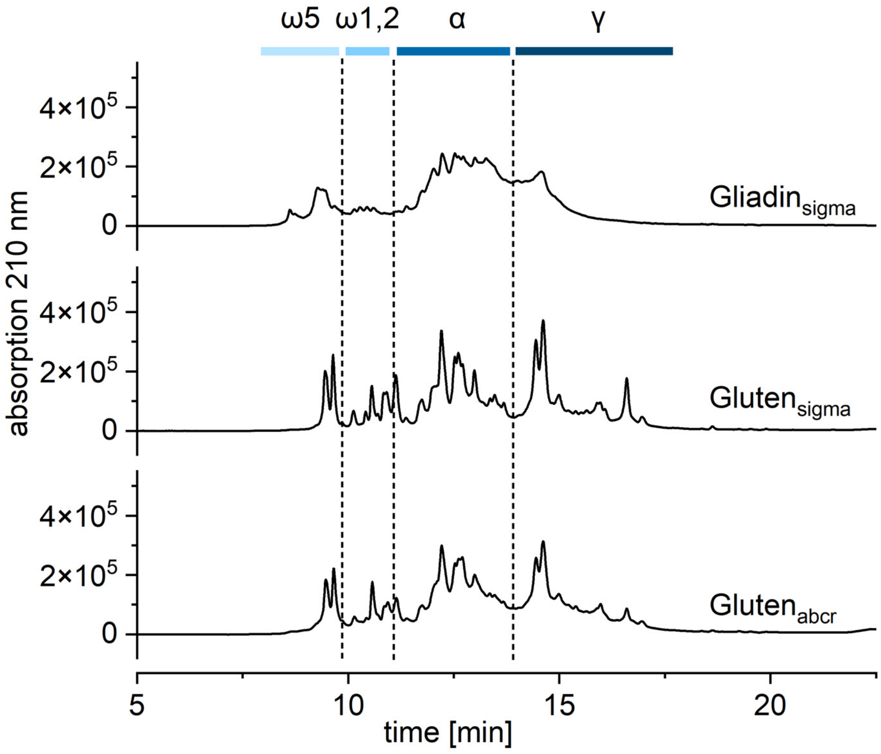

Figure S1. RP-HPLC chromatograms of the gliadin fraction of three commercially available gliadin and gluten materials obtained from Sigma-Aldrich (sigma) and abcr (abcr); batch 2. The integration times are marked as dotted lines separating the wheat gluten protein types ω5-gliadins (ω5), ω1,2-gliadins (ω1,2), α-gliadins (α) and γ-gliadins (γ).

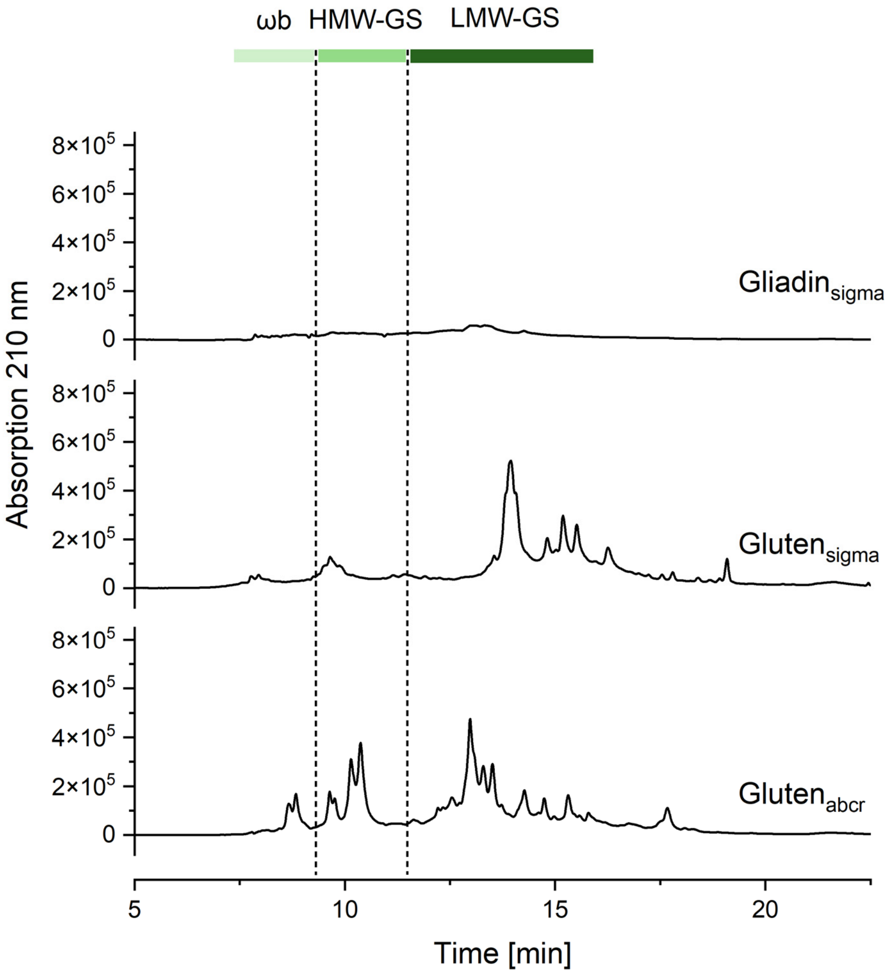

Figure S2. RP-HPLC chromatograms of the glutenin fraction of three commercially available gliadin and gluten materials obtained from Sigma-Aldrich (sigma) and abcr (abcr); batch 2. The integration times are marked as dotted lines separating the wheat gluten protein types ωb-gliadins (ωb), high-molecular-weight glutenin subunits (HMW-GS) and low-molecular-weight glutenin subunits (LMW-GS).

Table S1. Relative protein composition of three commercially available gliadin and gluten materials obtained from Sigma-Aldrich (sigma) and abcr (abcr) shown as proportions of the three different fractions albumins/globulins, gliadins and glutenins relative to total extractable proteins. Two batches each were compared (see Figure 4).

**Sample**	**Batch**	**Albumins/ Globulins**		**Gliadins**		**Glutenins**	
		Mean	SD	Mean	SD	Mean	SD
Gliadin$_{sigma}$	1	10.76^{BC}	0.17	68.33^{A}	1.60	20.90^{C}	1.76
	2	9.83^{BC}	1.14	71.81^{A}	4.44	18.36^{C}	3.31
Gluten$_{sigma}$	1	8.11^{C}	0.28	34.16^{B}	1.74	57.73^{A}	1.99
	2	4.88^{D}	0.24	35.87^{B}	6.16	59.25^{A}	6.36
Gluten$_{abcr}$	1	11.69^{B}	0.93	46.00^{B}	3.45	42.30^{B}	4.04
	2	15.70^{A}	1.32	40.98^{B}	5.47	43.32^{B}	4.20

[1] The values are given as means (n = 3) and different capital letters indicate significant differences between the samples in each column (one-way ANOVA, Tukey's post hoc test, $p < 0.05$). SD, standard deviation

Table S2. Relative molecular mass (Mr) distribution of three commercially available gliadin and gluten materials obtained from Sigma-Aldrich (sigma) and abcr (abcr). The ranges were divided according to the following molecular masses: (1) >66 kDa, (2) 66-29 kDa, (3) 29-12.4 kDa, (4) <12.4 kDa (see Figure 6).

**Sample**	**>66 kDa**		**29–66 kDa**		**12.4–29 kDa**		**<12.4 kDa**	
	Mean	SD	Mean	SD	Mean	SD	Mean	SD
Gliadin fraction								
Gliadin$_{sigma}$	21.26^{A}	0.68	10.16^{A}	0.014	29.45^{C}	0.15	39.12^{C}	0.51
Gluten$_{sigma}$	11.92^{B}	0.35	5.42^{C}	0.06	35.12^{A}	0.15	47.54^{A}	0.26
Gluten$_{abcr}$	20.20^{A}	0.09	8.62^{B}	0.04	30.86^{B}	0.10	40.32^{B}	0.12
reduced Gliadin fraction								
Gliadin$_{sigma}$	3.13^{A}	0.77	4.75^{B}	0.27	24.53^{C}	0.16	67.59^{A}	1.11
Gluten$_{sigma}$	1.45^{B}	0.13	4.32^{B}	0.09	50.87^{A}	0.36	43.36^{B}	0.15
Gluten$_{abcr}$	3.62^{A}	0.06	7.61^{A}	0.08	44.73^{B}	0.24	44.05^{B}	0.10
Gluten fraction								
Gliadin$_{sigma}$	14.81^{B}	0.36	6.99^{B}	0.10	27.30^{C}	0.11	50.91^{A}	0.41
Gluten$_{sigma}$	11.00^{C}	0.15	12.76^{A}	0.29	45.64^{A}	0.15	30.60^{B}	0.44
Gluten$_{abcr}$	17.05^{A}	0.93	13.02^{A}	0.57	38.93^{B}	0.47	30.99^{B}	0.98

[1] The values are given as means (n = 3) and different capital letters indicate significant differences between the samples in each column and each fraction (one-way ANOVA, Tukey's post hoc test, $p<0.05$). SD, standard deviation

Disclaimer/Publisher's Note: The statements, opinions and data contained in all publications are solely those of the individual author(s) and contributor(s) and not of MDPI and/or the editor(s). MDPI and/or the editor(s) disclaim responsibility for any injury to people or property resulting from any ideas, methods, instructions or products referred to in the content.
